# 中国嗜酸性粒细胞增多症诊断和治疗指南（2024版）

**DOI:** 10.3760/cma.j.cn121090-20231222-00334

**Published:** 2024-01

**Authors:** 中华医学会血液学分会白血病淋巴瘤学组

**Keywords:** 嗜酸性粒细胞增多症, 诊断, 治疗, 指南, Eosinophilia, Diagnosis, Treatment, Guideline

## Abstract

嗜酸性粒细胞增多症包括一组非血液系统（继发或反应性）和血液系统（原发或克隆性）疾病，该组疾病可能伴有器官受损。基于新的临床研究数据和对疾病分子遗传学解析结果，2022年世界卫生组织（WHO）和国际共识分型（ICC）对嗜酸性粒细胞增多性疾病的诊断和分型标准进行了更新。本指南是在《嗜酸性粒细胞增多症诊断和治疗中国专家共识（2017年版）》基础上的更新，旨在为我国血液学工作者提供一个规范的嗜酸性粒细胞增多症的诊断程序、实验室检查、诊断标准和治疗原则。

## Abstract (en)

The eosinophilias encompass a broad range of nonhematologic (secondary or reactive) and hematologic (primary or clonal) disorders with potential for end-organ damage. Based on new clinical data and increased understanding of disease molecular genetics, the World Health Organization (WHO) and the international consensus classification (ICC) has provided updated criteria and classifications for eosinophilic disorders in 2022. This guideline represents an update of Chinese expert consensus on the diagnosis and treatment of eosinophilia published in 2017 and aim to provide Chinese hematologist with clear guidance on management for eosinophilic disorders.

自嗜酸性粒细胞增多症诊断和治疗中国专家共识（2017年版）[1]颁布以来，对嗜酸性粒细胞增多症基于分子学的诊断分型和治疗有了许多新的认知，为了规范我国嗜酸性粒细胞增多症的临床诊治，由中华医学会血液学分会白血病淋巴瘤学组牵头, 参考国际最新诊治指南[2]–[7]，在广泛征求国内专家意见基础上，最终达成了嗜酸性粒细胞增多症的诊断程序、实验室检查、诊断标准和治疗原则等方面的共识，特制定了本指南。

一、定义和分类[2]–[7]

1. 嗜酸性粒细胞增多症（Eosinophilia）：外周血嗜酸性粒细胞绝对计数>0.5×10^9^/L。

2. 高嗜酸性粒细胞增多症（Hypereosinophilia, HE）：外周血2次检查（间隔时间>1个月）嗜酸性粒细胞绝对计数>1.5×10^9^/L和（或）骨髓有核细胞计数嗜酸性粒细胞比例≥20％和（或）病理证实组织嗜酸性粒细胞广泛浸润和（或）发现嗜酸性粒细胞颗粒蛋白显著沉积（在有或没有较明显的组织嗜酸性粒细胞浸润情况下）。

3. HE的分类：分为遗传性（家族性）HE（HE_FA_）、继发性（反应性）HE（HE_R_）、原发性（克隆性/肿瘤性）HE（HE_N_）和意义未定（特发性）HE（HE_US_）四大类。

（1）HE_FA_：发病机制不明，常见于儿童，有些患者同时伴有免疫缺陷，无HE_R_和HE_N_证据。

（2）HE_R_：主要可能原因有：①感染性疾病：寄生虫、病毒（如HIV、单纯疱疹病毒、HTLV-2）、真菌、细菌、分枝杆菌感染等；②过敏/变态反应性疾病：如哮喘、特应性皮炎、花粉症、过敏性胃肠炎等；③呼吸道疾病：Lǒeffler综合征、过敏性支气管肺曲霉菌病等；④心脏病：热带心内膜纤维化、嗜酸性粒细胞性心内膜心肌纤维化或心肌炎等；⑤皮肤病：特应性皮炎、荨麻疹、湿疹、大疱性类天疱疮、疱疹样皮炎、Gleich综合征等；⑥结缔组织/自身免疫性疾病：炎症性肠病、乳糜泻、嗜酸性肉芽肿性多血管炎、类风湿性关节炎、系统性红斑狼疮、结节性多动脉炎、结节病、系统性硬化症、干燥综合征、大疱性类天疱疮、IgG4相关疾病、嗜酸性粒细胞性筋膜炎等；⑦药物：阿司匹林、非甾体抗炎药、抗生素、药物反应伴嗜酸性粒细胞增多和全身症状综合征（DRESS）等；⑧肿瘤：实体瘤、淋巴瘤和急性淋巴细胞白血病（嗜酸性粒细胞为非克隆性）、系统性肥大细胞增多症（嗜酸性粒细胞为非克隆性）等；⑨免疫系统疾病：高IgE综合征、Omenn综合征、Wiskott-Aldrich综合征、IgA缺乏症等；⑩其他：急性/慢性移植物抗宿主病、实体器官排斥反应、木村病等。

（3）HE_N_：是指嗜酸性粒细胞起源于血液肿瘤克隆，定名为髓系和淋系肿瘤伴嗜酸性粒细胞增多症和酪氨酸激酶基因融合（Myeloid/lymphoid neoplasms with eosinophilia and tyrosine kinase gene fusions, M/LN-eo-TK）。根据受累基因不同，分为MLN伴PDGFRA重排、MLN伴PDGFRB重排、MLN伴FGFR1重排、MLN伴JAK2重排、MLN伴FLT3重排和MLN伴ETV6∷ABL1等6个亚型。

（4）HE_US_：查不到上述引起嗜酸性粒细胞增多的原发或继发原因。

4. HE相关的器官受损[1],[5]：器官功能受损，伴显著的组织嗜酸性粒细胞浸润和（或）发现嗜酸性粒细胞颗粒蛋白广泛沉积（在有或没有较显著的组织嗜酸性粒细胞浸润情况下）且有以下1条及以上：①纤维化（肺、心脏、消化道、皮肤和其他脏器组织）；②血栓形成伴或不伴栓塞；③皮肤（包括黏膜）红斑、水肿/血管性水肿、溃疡、瘙痒和湿疹；④外周或中枢神经病伴或不伴慢性或反复神经功能障碍。

5. 高嗜酸性粒细胞增多综合征（Hypereosinophilic syndrome，HES）：符合HE诊断标准，同时有HE相关的器官受损且需除外其他可作为主因导致器官受损的疾病。HES包括：①特发性HES，其中包括可独立诊断的淋巴细胞变异性HES（L-HES）（流式细胞术免疫表型分型证实CD3^+^、CD4^−^、CD8^−^或CD3^−^、CD4^+^或 CD3^+^、CD4^+^、CD7^−^的异常T淋巴细胞）；②原发性（肿瘤性）HES；③继发性（反应性）HES。

二、诊断程序[1]–[9]

通过仔细询问病史、查体，以及相关实验室检查，明确导致嗜酸性粒细胞增多症的可能原因，并评价可能的嗜酸性粒细胞相关终末器官受损或功能异常（图1）。

**图1:**
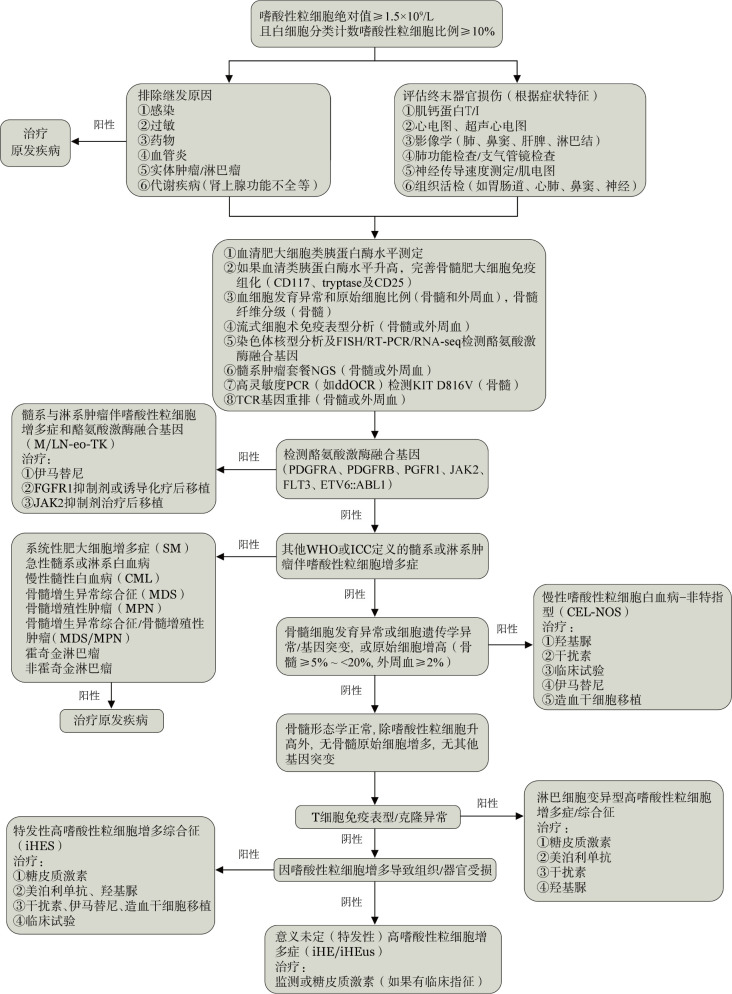
嗜酸粒细胞增多症诊断与治疗流程图

1. 询问病史时应仔细寻问有无过敏性疾病、有无皮疹或淋巴结肿大史、有无心肺和胃肠道症状。有无发热、盗汗、体重下降、瘙痒和酒精诱导的疼痛等体质性症状。详细询问旅游史，特别是有无热带地区旅游史。

2. 所有嗜酸性粒细胞增多症患者均应进行以下常规实验室检查：①全血细胞计数和外周血涂片分类计数；②常规生化检查，包括肝、肾功能，电解质和乳酸脱氢酶；③红细胞沉降率和（或）C反应蛋白；④血清VitB12。

3. 无症状且仅轻至中度嗜酸性粒细胞增多［嗜酸性粒细胞绝对计数在（0.5～1.5）×10^9^/L］患者，可以暂不进行进一步检查。

4. 有全身症状或持续性嗜酸性粒细胞增多（>1.5×10^9^/L），伴或不伴有可疑器官受损，首先应进行以下检查，确定或排除继发原因。

（1）考虑过敏原因：血清IgE，变应原特异的IgE，特异过敏症的皮肤针刺试验。

（2）考虑非过敏性皮肤原因：皮肤活检。

（3）考虑感染原因：大便寄生虫和虫卵镜检，可疑感染寄生虫的血清学试验，HIV和HTLV-2。

（4）考虑胃肠道原因：上胃肠道内镜，小肠镜或肛肠镜检查，血清淀粉酶，乳糜泻相关自身抗体的血清学检测。

（5）考虑结缔组织病：抗核抗体（ANA）或抗双链DNA抗体（dsDNA）等风湿抗体相关检查。

（6）考虑血管炎：抗中性粒细胞胞质抗体（ANCA），HBV、HCV、HIV、CMV和B19病毒的血清学检测。

（7）考虑呼吸疾病：影像检查（如肺部CT），纤维支气管镜。

5. 无明确继发原因且嗜酸性粒细胞增多（>1.5×10^9^/L）患者，应考虑血液系统恶性肿瘤伴克隆性嗜酸性粒细胞增多，为确定或排除可能疾病，应进行以下检查：①骨髓穿刺涂片分类计数；②骨髓活检组织切片病理细胞学分析；③FISH或RT-PCR检测FIP1L1::PDGFRA融合基因；④染色体核型分析；⑤血清肥大细胞类胰蛋白酶；⑥T细胞免疫表型分析±TCR基因重排；⑦如果染色体核型分析显示累及4q12（PDGFRA）、5q31-33（PDGFRB）、8p11-12（FGFR1）、9p24（JAK2）、9q34（ABL1）、13q12（FLT3），或其他酪氨酸激酶基因位点的染色体易位，则应采取RT-PCR或RNA测序方法（RNA-seq）确定相关融合基因。

6. 如果有可疑嗜酸性粒细胞增多所致的器官受损，应进行受累器官的评估。

（1）心脏评估：心电图、心肌肌钙蛋白和（或）N-末端原脑钠肽（NT-proBNP）测定与超声心动图和（或）心脏MRI（在心肌肌钙蛋白升高或有心脏损伤的临床特征情况下）有助于确定是否有心脏受累和（或）器官损伤。

（2）肺脏评估：肺功能检查、肺部影像学检查（如肺HRCT）、纤维支气管镜检查和支气管肺泡灌洗有助于确认患者肺部受累。

（3）外周和中枢神经系统评估：确认嗜酸性粒细胞诱导的周围神经病变需要做肌电图和神经活检。

（4）有耳鼻喉症状的患者应进行鼻窦炎、鼻息肉病和感音神经性听力损失评估。

（5）确认组织嗜酸性粒细胞增多和嗜酸性粒细胞导致的器官损伤需做器官活组织检查（皮肤、肺或肝活组织检查）并进行免疫组织化学染色检查（IHC）。胃肠道受累的患者应做胃、肠镜检查和相关黏膜活组织检查并做IHC（CD25、CD117和类胰蛋白酶）。确认皮肤受累伴嗜酸性粒细胞性筋膜炎需做深层皮肤活检和MRI。

（6）不明原因的血栓事件应记录为一种嗜酸性粒细胞相关的组织损害。

（7）有终末器官受损的患者，随访期间器官功能的监测频度依据器官受损的严重程度和广度和（或）嗜酸性粒细胞增多症的恶变来加以决定。

三、诊断与鉴别诊断

1. 引起继发性嗜酸性粒细胞增多症原发病的诊断参考相应诊断标准。

2. M/LN-eo-TK的诊断与分期[2]–[8]：

（1）确定疾病的程度：疾病是否累及骨髓、外周血或髓外疾病（extramedullary disease，EMD）或均有累及。

（2）疾病分期：根据骨髓和外周血相关检查，按现有标准[10]–[13]确定是慢性期（chronic phase）还是急变期（blast phase）。慢性期骨髓和外周血可表现为骨髓增殖性肿瘤（MPN）或骨髓增生异常综合征（肿瘤）/骨髓增殖性肿瘤［MDS（N）/MPN］，伴或不伴有嗜酸性粒细胞增多，骨髓中也可出现不典型肥大细胞增殖。急变期（原始细胞≥20％）骨髓和外周血可表现为急性髓系白血病（AML）、急性淋巴细胞白血病（ALL）和混合系别急性白血病（MPAL）。迄今尚无类似慢性髓性白血病（CML）加速期（骨髓或外周血原始细胞10％～19％）的界定。EMD等同为急变期，EMD可表现为髓系肉瘤、T细胞/B细胞淋巴瘤，或髓系/T或B淋巴细胞混合系别急变期疾病。EMD可以单独存在或与骨髓或外周血受累的慢性期或急变期疾病共存，EMD受累系别可以与骨髓或外周血受累系别不同。

（3）确定骨髓或外周血和EMD受累系别：采用免疫表型分析（流式细胞术免疫表型分析和/或IHC）确定骨髓或外周血和EMD受累系别。

3. 特发性高嗜酸性粒细胞增多综合征（iHES）诊断标准[3]–[5]：①除外以下情况：反应性嗜酸性粒细胞增多症；淋巴细胞变异型嗜酸性粒细胞增多症（产生细胞因子，免疫表型异常的T细胞亚群）；慢性嗜酸性粒细胞白血病-非特指（CEL-NOS）；WHO标准可确诊的髓系肿瘤（如MDS、MPN、MDS/MPN、AML）伴嗜酸性粒细胞增多；M/LN-eo-TK。②嗜酸性粒细胞绝对计数>1.5×10^9^/L，必须至少持续6个月，且必须有组织受损。如果没有组织受损，则诊断特发性高嗜酸性粒细胞增多症。

4. iHES与CEL-NOS的鉴别：CEL-NOS归属骨髓增殖性肿瘤，可有非特异性克隆细胞遗传学或分子异常或原始细胞增加（外周血>2％或骨髓>5％），骨髓细胞形态有明显的巨核细胞发育异常，可同时伴有粒系和（或）红系发育异常，MF-2或MF-3纤维化。iHES与CEL-NOS的主要鉴别要点见表1。

**表1:** 特发性高嗜酸粒细胞增多综合征（iHES）与慢性嗜酸粒细胞白血病-非特指（CEL-NOS）的鉴别诊断要点

iHES		CEL-NOS
定义	持续嗜酸性粒细胞增高^a^，伴嗜酸性粒细胞（非反应性或肿瘤性）所致的组织受损/功能不全	一种伴嗜酸性粒细胞显著且持续增殖的骨髓增殖性肿瘤。骨髓和外周血原始细胞<20%，不符合AML诊断标准
外周血	持续的HE（嗜酸性粒细胞绝对计数≥1.5×10^9^/L且白细胞分类计数嗜酸性粒细胞比例≥10%）	持续的HE（嗜酸性粒细胞绝对计数≥1.5×10^9^/L且白细胞分类计数嗜酸性粒细胞比例≥10%）
器官受损和（或）功能不全	是诊断必备条件	非诊断必备条件
病因	无反应性、自身免疫或肿瘤性疾病或可导致HE的疾病的证据	一种伴有克隆性嗜酸性粒细胞增殖的髓系肿瘤
需要除外情况	除外反应性和肿瘤性HE，包括淋巴细胞变异性HES	没有酪氨酸激酶基因融合，包括BCR∷ABL1，ETV6∷ABL1，PDGFRA、PDGFRB、FGFR1、JAK2、FLT3融合，不符合任何AML、CMML、SM的其他诊断标准
骨髓	除嗜酸性粒细胞增高外，骨髓形态均在正常范围	骨髓增生程度增高伴巨核细胞发育异常，伴或不伴有包括嗜酸性粒细胞在内的其他系别细胞的发育异常，常有显著纤维化以及嗜酸性粒细胞浸润或骨髓中的原始细胞≥5%和（或）外周血原始细胞≥2%
分子遗传学	无分子遗传克隆异常，可伴有潜质未定的克隆性造血（CHIP）	有确定的克隆性细胞遗传学异常和（或）基因突变

注 AML：急性髓系白血病；HE：高嗜酸性粒细胞增多症；^a^最好至少持续6个月，但对于出现器官损伤/功能不全的患者，需要立即治疗，持续4周或间隔时间为2周的2次可能就足够了。CEL-NOS的诊断：嗜酸性粒细胞增多持续时间优选3个月，在需要治疗的患者中，最少1个月，至少2次

5. M/LN-eo-TK与Ph-like ALL的鉴别：JAK2与其他伴侣基因的融合，例如t（5；9）（q14.1；p24.1）/SSBP2::PAX5和PAX5::JAK2通常是BCR∷ABL1样B-ALL，而不是M/LN-eo-TK，二者的主要鉴别手段是至少选一个JAK2探针采用FISH方法证实嗜酸性粒细胞是否有JAK2基因受累，如阳性则诊断M/LN-eo-TK，阴性则诊断Ph-like ALL。

四、治疗[2]–[8]

继发性嗜酸性粒细胞增多症主要是针对原发病的治疗。原发性和特发性嗜酸性粒细胞增多症一般以重要器官受累和功能障碍作为主要治疗指征。由于外周血嗜酸性粒细胞绝对计数不一定与终末器官受损呈正比，因此，如果没有明确的器官受累和功能障碍，尚无是否需要治疗以及何时开始治疗的共识。嗜酸性粒细胞增多症治疗的目的是降低嗜酸性粒细胞绝对计数和减少嗜酸性粒细胞介导的器官功能受损。

1. 紧急处理：当有严重的或致命性器官受累，特别是心脏和肺，应进行紧急处理。首选静脉输注甲泼尼龙1 mg·kg^−1^·d^−1^，或口服泼尼松0.5～1.0 mg·kg^−1^·d^−1^。如果极度的嗜酸性粒细胞增多，应同时给予别嘌呤醇。1～2周后逐渐缓慢减量，2～3个月减量至最少维持剂量。

2. M/LN-eo-TK的治疗：

（1）PDGFRA和PDGFRB重排患者的治疗：慢性期患者首选伊马替尼，起始剂量为100 mg/d，如疗效不佳，可加大剂量至400 mg/d，直至达完全临床、血液学和分子生物学缓解[3],[7]，[14]-[15]。检测外周血细胞计数和白细胞分类计数，如果有器官受损应同时评估靶器官功能，治疗3个月后进行细胞遗传和分子学疗效评估，如达到完全血液学、细胞遗传和分子学缓解，则进入维持治疗阶段。维持治疗方案尚无共识，可继续维持原剂量，或改为隔日1次或每周1次给药，以维持临床完全缓解及嗜酸性粒细胞计数在正常范围。急变期患者采用伊马替尼（100～400 mg/d）联合原发肿瘤治疗方案作为造血干细胞移植（HSCT）前桥接治疗，如果适合HSCT应尽快进行HSCT。已有PDGFRA基因突变（常见突变为T674I和D842V）和DGFRB基因突变（如C843G）导致伊马替尼耐药的报道[3],[7]，[16]-[18]。

（2）FGFR1重排患者的治疗：首选参加临床试验，如无合适临床试验，慢性期患者可选择培米替尼（pemigatinib）或米哚妥林、普纳替尼、奥雷巴替尼，急变期采用培米替尼（pemigatinib）或米哚妥林、普纳替尼、奥雷巴替尼联合AML或ALL样化疗作为HSCT前桥接治疗，如果适合HSCT应尽快进行HSCT[3],[7],[19]–[20]。

（3）ABL1和FLT3重排患者的治疗：首选参加临床试验，如无合适临床试验，ABL1重排慢性期患者可选择达沙替尼、尼洛替尼，或阿西米尼（asciminib）、博舒替尼、伊马替尼和普纳替尼，FLT3重排患者可选用吉瑞替尼、米哚妥林、索拉非尼和舒尼替尼，急变期采用上述药物联合AML或ALL样化疗作为HSCT前桥接治疗，如果适合HSCT应尽快进行HSCT[3],[7],[21]。

（4）JAK2重排患者的治疗：首选参加临床试验，如无合适临床试验，慢性期患者可选用芦可替尼或菲卓替尼治疗，急变期采用上述药物联合AML或ALL样化疗作为HSCT前桥接治疗，如果适合HSCT应尽快进行HSCT[3],[7],[22]。

3. 意义未定（特发性）嗜酸性粒细胞增多症的治疗[3]–[7],[23]：

（1）一线治疗首选泼尼松1 mg·kg^−1^·d^−1^口服，1～2周后逐渐缓慢减量，2～3个月减量至最少维持剂量[24]。若减量过程中病情反复，至少应恢复至减量前用药量。完全和部分缓解率为65％～85％。治疗1个月后如果嗜酸性粒细胞计数>1.5×10^9^/L或最低维持剂量>10 mg/d，应改用二线治疗。

二线治疗药物选择包括：①伊马替尼400 mg/d，4～6周后无效则停用；②干扰素[3],[7]，剂量选择尚无共识，一般为100～500 IU·m^−2^·d^−1^，一般需数周后方可起效；③环孢素A，剂量文献报道150～500 mg/d不等；④硫唑嘌呤，推荐起始剂量为1～3 mg·kg^−1^·d^−1^，肝、肾功能不全患者应选用较低起始剂量，依患者临床和血液学反应调整剂量；⑤羟基脲0.5～3.0 g/d，可单用或与干扰素联合使用；⑥单克隆抗体，如Mepolizumab、reslizumab和Alemtuzumab等。

（2）支持及手术治疗：对于白细胞和嗜酸性粒细胞计数较高患者，可进行细胞单采术，抗凝或抗血小板治疗具有预防血栓作用；换瓣手术可能使心脏瓣膜有损害的患者获益。

五、疗效判断标准

最近MLN国际工作组以培米替尼治疗FGFR1重排的M/LN-eo-TK的Ⅱ期临床试验FIGHT-203试验方案为基础提出了一个M/LN-eo-TK的疗效判断标准[24]，但该标准尚待以后前瞻性临床试验来加以确证。
